# Problems with studying directional natural selection in humans

**DOI:** 10.18699/VJGB-23-79

**Published:** 2023-10

**Authors:** S.V. Mikhailova

**Affiliations:** Institute of Cytology and Genetics of the Siberian Branch of the Russian Academy of Sciences, Novosibirsk, Russia

**Keywords:** natural selection, Homo sapiens, fertility, adaptation, polygenic index, genome-wide association study, естественный отбор, Homo sapiens, фертильность, адаптация, полигенный индекс, полногеномный анализ ассоциаций

## Abstract

The review describes the main methods for assessing directional selection in human populations. These include bioinformatic analysis of DNA sequences via detection of linkage disequilibrium and of deviations from the random distribution of frequencies of genetic variants, demographic and anthropometric studies based on a search for a correlation between fertility and phenotypic traits, genome-wide association studies on fertility along with genetic loci and polygenic risk scores, and a comparison of allele frequencies between generations (in modern samples and in those obtained from burials). Each approach has its limitations and is applicable to different periods in the evolution of Homo sapiens. The main source of error in such studies is thought to be sample stratification, the small number of studies on nonwhite populations, the impossibility of a complete comparison of the associations found and functionally significant causative variants, and the difficulty with taking into account all nongenetic determinants of fertility in contemporary populations. The results obtained by various methods indicate that the direction of human adaptation to new food products has not changed during evolution since the Neolithic; many variants of immunity genes associated with inflammatory and autoimmune diseases in modern populations have undergone positive selection over the past 2–3 thousand years owing to the spread of bacterial and viral infections. For some genetic variants and polygenic traits, an alteration of the direction of natural selection in Europe has been documented, e. g., for those associated with an immune response and cognitive abilities. Examination of the correlation between fertility and educational attainment yields conflicting results. In modern populations, to a greater extent than previously, there is selection for variants of genes responsible for social adaptation and behavioral phenotypes. In particular, several articles have shown a positive correlation of fertility with polygenic risk scores of attention deficit/hyperactivity disorder.

## Abstract (ru)

В обзоре описаны основные методы оценки направленного отбора в популяциях человека. В их числе биоинформатический анализ последовательностей ДНК, основанный на выявлении неравновесия по сцеплению и отклонения от случайного распределения частот генетических вариантов; демографические и антропометрические исследования, заключающиеся в поиске корреляции рождаемости с фенотипическими признаками; полногеномные оценки ассоциаций фертильности с генетическими локусами и полигенными индексами, а также сравнение частот аллелей между поколениями (как в современных выборках, так и в полученных из захоронений ДНК). Каждый из этих подходов имеет свои ограничения и применим к разным периодам эволюции Homo sapiens. Основными источниками ошибок в таких исследованиях считаются стратификация выборок, ограниченное число исследований на неевропеоидном населении, невозможность полного сопоставления найденных ассоциаций и функционально значимых каузативных вариантов, а также сложность учета всех негенетических факторов, определяющих фертильность в современных популяциях. Полученные с помощью разных методов результаты свидетельствуют о том, что направление адаптации человека к новым для него пищевым продуктам не меняется в ходе эволюции с неолита; многие из вариантов генов иммунитета, ассоциированных в современных популяциях с воспалительными и аутоиммунными заболеваниями, подверглись положительному отбору в период последних 2–3 тыс. лет в связи с распространением бактериальных и вирусных инфекций. По некоторым генетическим вариантам и полигенным признакам показана смена направления естественного отбора на территории Европы, среди них связанные с иммунным ответом и когнитивными способностями. Анализ корреляции фертильности и уровня образования дает противоречивые результаты. В современных популяциях в большей степени, чем ранее, наблюдается отбор по вариантам генов, отвечающих за социальную адаптацию и поведенческие фенотипы. В том числе в нескольких работах показана положительная корреляция фертильности с полигенными индексами синдрома дефицита внимания/гиперактивности

## Introduction

It has been shown that the prevalence of some diseases is increasing in human populations around the world. These include obesity (along with related disorders: type 2 diabetes mellitus and coronary heart disease), attention deficit/hyperactivity disorder (ADHD), autism spectrum disorders, and allergic diseases (Charpin, Gouitaa, 2001; Saklayen, 2018; Zeidan et al., 2022; Wolf et al., 2023). This phenomenon requires new approaches in medicine, social services, education, leisure, and nutrition of children and adolescents

At the same time, there is no consensus on the causes of the observed changes; numerous studies are being conducted to find a relation of the diseases with the lifestyle, maternal stress, and environmental pollution. The human environment has changed dramatically in the last century with the advent of new chemical compounds, the growth of anthropogenic pollution, and the progress of medicine, including reproductive technologies. Urbanization has led to an increase in population density and social stress (Suvorov, 2021). The demographic transition has caused the aging of populations; the extended life expectancy affected the incidence of metabolic disorders and cardiovascular pathologies. In addition, research on the contribution of human genetics to the increased disease incidence remains relevant. Standard approaches of medical genetics aimed at finding associations of genetic variants with diseases do not allow to answer the question of the direction of selection in modern people; this is because (i) these diseases – especially in the postreproductive period – are not directly related to the number of children an individual has, (ii) homozygous or heterozygous carriage of a genetic variant can affect fitness in different ways, and (iii) most diseases are multifactorial, and the same is true for personality traits. Currently, several techniques are used to assess natural selection in human populations.

## Bioinformatic methods

The analysis of DNA sequences in large human cohorts allows to estimate the direction of natural selection over the past millennia. For this purpose, methods, algorithms, and software packages have been developed based on estimates of the frequency of polymorphic variants and linkage disequilibrium in the areas of their location (Grossman et al., 2013; Field et al., 2016; Palamara et al., 2018; Speidel et al., 2019; Abondio et al., 2022). It is assumed that (a) longer haplotypes (areas that have not undergone recombination) in any region of the genome, (b) increased frequencies of derived alleles in adjacent loci, (c) a greater difference in allele frequencies between populations than could be expected during genetic drift, and (d) a higher rate of substitutions at each position (calculated from a genomic alignment of 100 vertebrate species) may be indicative of directional selection in the genomic region in question

In a study performed on the 1000 Genomes database, 412 regions with signs of directional selection were analyzed (Grossman et al., 2013). These regions proved to be enriched with coding variants: 235 regions containing one or more protein-coding genes and 177 regions without genes encoding known proteins were found. In addition, 48 genes of long intergenic noncoding RNAs were revealed there. Among the 33 genes containing the most highly ranked single-nucleotide variants (SNVs), there are SCL24A5 and MATP (associated with reduced skin pigmentation), EDAR (affecting the formation of hair, sweat glands, and teeth), ARHGEF3 (affecting bone mineral density), BTLA (associated with rheumatoid arthritis), CTNS (affecting cysteine metabolism), ITPR3 (associated with type 1 diabetes mellitus), innate-immunity receptor gene TLR5, ITGAE (involved in cell adhesion and lymphocyte activation), and AP4B1 (associated with cerebral palsy). A total of 11 loci were associated with height and pigmentation, and 79 with a predisposition to infectious and autoimmune diseases. The SLC24A5 gene, in addition to pigmentation, determines resistance to leprosy. Genes ALMS1, CCR9, CXCR4, and VDR are also located in the loci associated with resistance to infections and having signs of selection.

Another study, performed on 2,478 people of various backgrounds from the 1000 Genomes database, has identified several genomic regions with positive selection for multiallelic traits, including red and white blood cell counts, hair color, and the body mass index (BMI) (Speidel et al., 2019). Among the targets of positive selection, enrichment with SNVs in functional regions of the genome was observed in that paper. Signs of positive selection were found, in the major histocompatibility complex (MHC) locus and genes LCT (associated with lactose tolerance), EDAR, EDARADD, HERC1 (associated with syndromic mental retardation), and ATXN2 (associated with type 1 diabetes mellitus, obesity, and hypertension). In the same article, the proposed method of looking for signs of positive selection was applied to British Biobank data, resulting in a signal for SNVs associated with lighter hair in Europeans. The most significant signal identified is related to SNVs associated with a reduced BMI in white Americans. The largest number of loci with signs of selection was found in Europeans, whereas East Asians have the least number of signals (presumably owing to the “bottleneck” effect in their history) (Speidel et al., 2019).

In an examination of data from the British Biobank, 12 directional selection signals were detected, including those located near immunity genes (TLR1-6-10, HLA, IGHG, STAT4, MUC5B, FAM19A5, and ANXA), near the genes that determine pigmentation (GRM5 and MC1R), and near LCT (Palamara et al., 2018). As a result of the analysis of 3,195 genomes from British project UK10K, signs of selection over the past 2–3 thousand years have been found in MHC locus and in LCT and WDFY4 (associated with activation of T cells during viral infections) genes as well as in the genes responsible for skin and hair pigmentation (KITLG, OCA2/HERC2, ASIP, and SLC24A4). Polygenic selection has been identified for variants predisposing to higher growth and lower total cholesterol in both sexes and to a reduced BMI in males (Field et al., 2016). By the same method applied to data of the British Biobank, other researchers (Song et al., 2021) have analyzed selection signals for 870 disease-associated polygenic traits and 15 nondisease- associated phenotypic traits. It was noted that during the last 2–3 thousand years, 88 % of polygenic traits have undergone selection. The highest scores were shown by genes that determine the ease of skin tanning and lighter hair. Selection for most disease-related loci, including those associated with autism spectrum disorders and elevated cholesterol, was negative; an exception was genes predisposing to Crohn’s disease and ADHD. For polygenic scores of intelligence and insomnia, a change in the direction of adaptation was predicted at ~133 generations ago.

Twenty-nine genetic loci with signs of directional selection were identified in biological samples from the Japan Biobank (Yasumizu et al., 2020). The highest statistical significance was registered for the alcohol dehydrogenase (ADH ) gene cluster and for genes CIAO2A (metal ion binding), MYOF (cell membrane regeneration), GRIA2 (glutamate receptor), and ASAP2 (vesicular transport). That work also confirmed previously reported selection pressure on EDAR genes and on the MHC gene cluster in the Japanese population.

Several studies have assessed the evolution of genes associated with inflammatory diseases separately. In an evaluation of the length of haplotypes containing 588 SNVs associated with 10 inflammatory diseases in European populations, signs of relatively recent (within the last 1,200–2,600 years) positive selection were identified in 21 loci, while variants associated with diseases, not protective alleles, were being selected (Raj et al., 2013). As a result of a comparison of several common multifactorial diseases and phenotypic traits in terms of the number of SNVs under positive selection associated with them, most of these SNVs were implicated specifically in inflammatory diseases. The genetic variants found are mainly involved in the molecular pathways participating in the activation of T helper 17 (Th17) lymphocytes (STAT1, STAT3, STAT5, IRF1, CSF2, IL2, IL3, IL12A, IL2RA, and SOCS1) (Raj et al., 2013). On data from the Estonian Biobank (2,300 wholegenome sequences), a search for signs of directional selection was performed at 535 loci associated with 21 autoimmune diseases. As a result, 153 loci showed signs of selection, while 29 of them were found to be selected due to linkage with other variants (Pankratov et al., 2022). The largest number of loci found in this work was associated with leukocyte activation and cytokine synthesis.

The search for genetic variants that have been under selection is difficult because a substantial number of identified genome loci with signs of directional selection have been found in intergenic regions (Grossman et al., 2013; Yasumizu et al., 2020), for which no functional significance can be predicted. As a consequence, there is no answer to the question whether this effect is due only to the insufficient level of knowledge about noncoding regions of the genome or to the inaccuracy of the algorithms used.

## Comparison of genomes of ancient and modern humans

The accumulation of data on DNA sequences from human remains from burial sites makes it possible to compare them with genotypes of modern humans. A comparison of genotypes among people who lived at different times in the same geographic area is of particular interest.

When comparing ancient and modern genomes of people from Britain, investigators found seven loci to have signs of directional selection over the past 4,500 years (Mathieson, Terhost, 2022). Most of them are associated with vitamin D or calcium metabolism. It was demonstrated that the strength of selection for individual loci has changed over time, suggesting that some factors have appeared that have softened it. Among the 28 complex anthropometric and metabolic traits analyzed in that work, evidence of polygenic selection was detected only for skin pigmentation.

Polymorphic sites in innate-immunity genes associated with predisposition to mycobacterial infections (SLC11A1, MBL2, TLR2, P2RX7, IL10, and TNFA) have been studied in remains of 151 people in time series data (1st–18th centuries AD) from Northern and Eastern Poland (Lewandowska et al., 2018). This DNA analysis indicated that genetic drift has played the main role in the evolution of people in this region; however, two SNVs (rs17235409 of the divalent cation transporter SLC11A1 gene and rs1800896 of interleukin IL10 gene) manifested signs of nonrandom evolution.

In a collection of 1,013 genomes of Europeans born from the Mesolithic Age to the Middle Ages, the frequency of rs34536443 of the TYK2 gene has been estimated (Kerner et al., 2021). This gene encodes a tyrosine kinase that is involved in signal transduction from cytokine receptors. The minor allele of rs34536443 has previously been shown to be associated with susceptibility to tuberculosis. In that work (Kerner et al., 2021), it was found that this rare allele emerged as a single mutational event during the Early Neolithic ∼8,500 years ago on the Anatolian Peninsula, and then spread to Central Europe, where its frequency had remained within 3 % until ~5,000 years ago. In the Bronze Age, the frequency peaked (10 %) ~3,000 years ago, and after the Iron Age, it began to decline sharply until it reached 2.9 %. The observed changes in the frequency of rs34536443 are associated with the spread of tuberculosis in Europe. rs1800562 of the HFE gene (iron metabolism regulator) was also investigated by those authors (Kerner et al., 2021). Its maximum frequency in Europe (~10 %) was reached in the Middle Ages, and then it decreased in Europe to an average of 4 %.

Other work researchers have explored 827 ancient samples of European origin (from 25,000 years BC to the present) (Kuijpers et al., 2022). In accordance with the available data on genome-wide polygenic risk scores of inherited traits in Europeans, the genomes were compared at different time intervals. It was demonstrated that after the Neolithic, height and intelligence increased in the European population, skin pigmentation diminished, and the risk of coronary heart disease went up due to decreasing concentrations of high-density lipoprotein cholesterol. It was suggested that the latter trend is related to cognitive functions because variations in lipoprotein levels are associated with intelligence, learning, and memory. To identify the loci that were selected during the bubonic plague epidemic (Black Death) in Europe (1,347–1,351 AD), an association analysis has been performed on immunity genes in 206 DNA samples originating from burials of two European populations before, during, and after this epidemic (London: years ~1,000–1,250 and 1,350–1,539, and Denmark: ~850–1,350 and ~1,350–1,800) (Klunk et al., 2022). Four positively selected loci (rs2549794, which affects ERAP2 mRNA splicing, and three SNVs in noncoding regions: rs11571319, rs17473484, and rs1052025) proved to be common between British and Danish burials. It was shown that genes located near these SNVs (ERAP1, ERAP2, LNPEP, CTLA4, ICOS, TICAM2, and TMED7) are differentially expressed in human macrophages in response to Yersinia pestis infection.

An analysis of 187 polygenic traits in three sets of ancient human genomes (from 8,000–4,200, ~14,000–3,400, and ~45,000–7,000 years ago) indicates that in Near East genomes, selection signals for tan-determining genes varied depending on latitude; signs of positive selection were observed at low latitudes, and signs of negative one at high latitudes (Song et al., 2021). Positive selection signals were also demonstrated in that work for 13 disease loci, including Crohn’s disease, atopic dermatitis, and periodontitis.

During an analysis of a study population consisting of the 1000 Genomes database and 230 remains of inhabitants of Eurasia (dated from 6,500 to 1,000 BC), the strongest selection signal was detected near the LCT gene (rs4988235) (Mathieson et al., 2015). Two other independent signals were found: near FADS1 (rs174546) and DHCR7/NADSYN1 (rs7940244). FADS1 and FADS2 are fatty acid desaturases taking part in the synthesis of long-chain polyunsaturated fatty acids from short precursors; variants at this locus correlate with plasma fatty-acid concentrations. The most statistically significant SNV at this locus (rs174546) is associated with a reduced level of triglycerides. 7-Dehydrocholesterol reductase (encoded by the DHCR7 gene) participates in the metabolism of cholesterol and vitamin D. Additionally, directional selection was documented for the MHC and TLR1-6-10 immunity gene loci, genes HERC2 and SLC45A2 (responsible for pigmentation), loci near genes ATXN2, GRM5 (glutamate receptor), ZKSCAN3 (transcriptional regulation), and SLC22A4 (organic cation transporter).

The limitations of this approach to the analysis of directional selection are as follows: data from burial sites are fragmentary, and the observed alterations in genomes can be due to migrations from regions where the genetic pool was formed, for example, under the influence of bottleneck effects (Kerner et al., 2021) and assortative mating (Mills, Mathieson, 2022). In the work performed on Estonian Biobank data and on the Allen Ancient DNA Resource (AADR) V44.3 (Marnetto et al., 2022), it was shown that in a mixed population formed from previously isolated populations of pastoralists, huntergatherers, and farmers, some phenotypes are associated with the carriage of DNA sequences specific for ancestral populations in certain regions of the genome. Among other things, it was demonstrated that blood cholesterol levels among modern Estonians are positively correlated with the similarity of their genomes to those of carriers of the Yamnaya culture at loci associated with cholesterol levels. Thus, during the formation of a population, the observed difference in allele frequencies relative to an ancestral population may not necessarily indicate directional selection.

## Estimation of the number of children in carriers of different phenotypes and genotypes

In addition to changes in the prevalence of genetic variants or phenotypic traits over time, the presence of directional natural selection is indicated by differing numbers of offspring in carriers of certain genotypes or traits. After the demographic transition that has taken place in many countries and has featured a decline of infant and child mortality with a simultaneous decrease in fertility, it is the estimation of the number of children that is the most accurate method for researching modern directional selection in human populations. Cross-sectional studies on populations of young people do not reflect the real number of offspring because there is a tendency to postpone the birth of the first and hence subsequent children (Balbo et al., 2013). In an analysis of a sample older than 50 years, dead individuals, which nevertheless had offspring, fall out of sight. Therefore, long-term studies are the most promising, especially those that enable a comparison of successive generations. The most commonly used indicators of fertility are (i) the total number of children ever born (NEB) in women over 45 and in men over 55, (ii) the age at first birth, which shows a strong inverse correlation with NEB (Tropf et al., 2015; Kong et al., 2017; Sanjak et al., 2018; Arkhangelskiy et al., 2020), (iii) relative reproductive success (the number of children per individual divided by the average number of children per individual in the population), and (iv) childlessness.

Demographic and anthropometric studies

Fertility is the subject of demographic research. Special attention is paid to problems of population reproduction in countries that have made the demographic transition because the observed decline of the birth rate leads to the “aging” of the population, to a decrease in the proportion of working-age people, and as a result, to a decline of economic growth rates. Demographers seek to predict population size and fertility and to detect its possible association with socioeconomic factors, which would allow to regulate population reproduction. Thus, demography essentially investigates the association of complex phenotypic traits with natural selection in a population.

A search for relations between fertility and income, educational attainment, religiosity, and anthropometric data has yielded mixed results (Table 1).

**Table 1.**
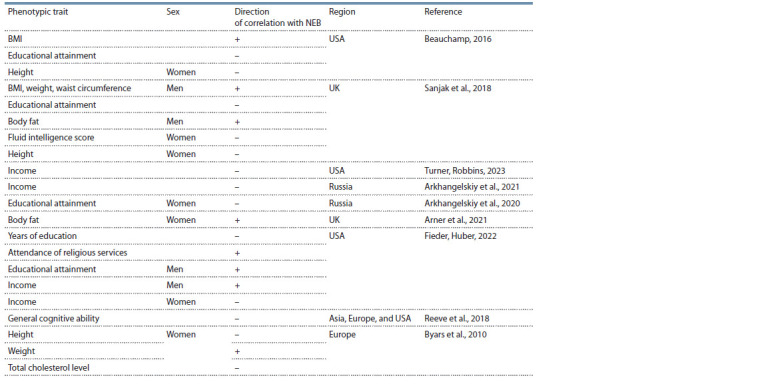
Described correlations of the number of children ever born (NEB) with demographic and anthropometric characteristics, if sex is not specified: for both sexes

Some articles point to an inverse correlation between the NEB and the income of individuals and households (Arkhangelskiy et al., 2021; Turner, Robbins, 2023), and others have revealed correlations of different directions when men are compared with women (Fieder, Huber, 2022); it has also been noted that after the division of a cohort into subgroups by income level, the direction of the correlation changes (Cohen et al., 2013; Arkhangelskiy et al., 2021). Demographers pay special attention to the analysis of correlations between the birth rate and educational attainment. In the vast majority of papers, indicators of education and cognitive abilities show an inverse correlation with the number of offspring (Beauchamp, 2016; Reeve et al., 2018; Sanjak et al., 2018; Arkhangelskiy et al., 2020; Fieder, Huber, 2022) (see Table 1). Of note, among men, educational attainment correlates with income, the above effect may change (Fieder, Huber, 2022). A survey of 9,452 women from 27 EU countries indicates that the estimated NEB is higher among women with higher education, but this study deals with intentions, not actually born offspring (Testa, 2014).

Anthropometric studies have uncovered a slow change in some parameters in populations. For example, since the 1950s, there has been a 0.5 % increment in the number of cases of fetal disproportion during childbirth, and this phenomenon is associated with a weakening of natural selection as a result of the massive use of cesarean section (Mitteroecker et al., 2016). A positive correlation of the NEB with physical characteristics has been documented, including weight, the BMI, and body fat (Byars et al., 2010; Beauchamp, 2016; Sanjak et al., 2018; Arner et al., 2021) (see Table 1). Modern data on selection for height contradict those obtained from ancient specimens; it has been reported that the number of children is negatively correlated with females’ height (Byars et al., 2010; Beauchamp, 2016). The Framingham two-generation study revealed reductions in total cholesterol and systolic blood pressure in a European population (Byars et al., 2010).

Nevertheless, the NEB depends on historical, cultural, economic, and social environments (for example, the availability of contraceptives and child care) (Barban et al., 2016). In demographic research, it is often impossible to establish causal relations between observed phenomena and to take into account all nongenetic factors, and this state of affairs makes it difficult to employ such data. In this regard, proposals are being made for the integration of genetic and demographic studies (Hugh-Jones, Abdellaoui, 2022).

Analysis of the association of fertility with genetic markers

The NEB has one of the highest levels of polygenicity of any trait (Mathieson et al., 2023). At the same time, NEB heritability in different articles is estimated at 14–46 % (Barban et al., 2016). In a sample of women from the UK and the Netherlands, it has been shown that up to 10 % of the NEB variance is determined by common genetic variants (Tropf et al., 2015). The main way to assess the effect of the genotype on fertility is a genome-wide analysis of the association of fertility rates in populations of modern people. The results of such research are given in Tables 2 and 3.

**Table 2.**
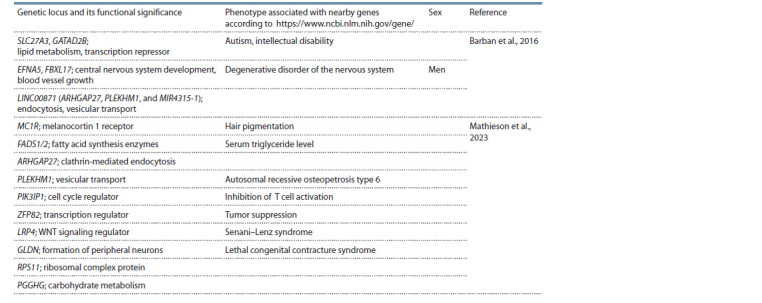
Genome-wide associations identified for the total number of children ever born, if sex is not specified: for both sexes

**Table 3.**
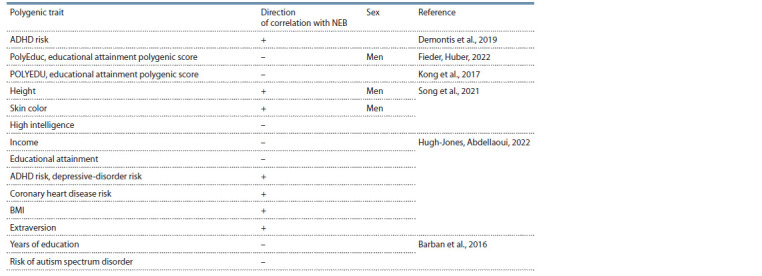
Polygenic associations found for the number of children ever born (NEB), if sex is not specified: for both sexes

A meta-analysis of NEB genome-wide association studies (343,072 people) has revealed three main loci: rs10908474 (near genes SLC27A3 and GATAD2B), rs13161115 (between genes EFNA5 and FBXL17), and rs2415984 in the intron of long intergenic noncoding RNA gene LINC00871 (the strongest signal). An additional study identified a locus near rs2415984 containing genes ARHGAP27, PLEKHM1, and MIR4315-1. An analysis of potential functional significance of these variants indicates linkage disequilibrium of rs13161115 with a methylation site near the EFNA5 gene (Barban et al., 2016) (see Table 2). For the loci associated with age at first birth, an inverse correlation with educational attainment has been shown. The NEB polygenic score inversely correlates with the number of years of education (see Table 3). Most of the correlations found in the above meta-analysis for the NEB and age at first birth polygenic scores are related to behavioral and reproductive phenotypes.

A recent genome-wide search for NEB and childlessness associations (785,604 Europeans) yielded 43 genomic loci associated with age of puberty, age at first birth, sex hormone regulation, endometriosis, and age at menopause (Mathieson et al., 2023) (see Table 2). Among them, 28 are common between men and women and six are gender-specific to the NEB; nine, including a gender-specific one, are associated with childlessness. rs12949256 of ARHGAP27 (p.Ala117Thr) is associated with a higher NEB but a shorter reproductive period. NEB-associated coding variants were found in genes PIK3IP1 (rs2040533, p.Thr251Ser), ZFP82 (rs17206365, p.Leu59Met), and LRP4 (rs6485702, p.Ile1086Val). A comparison of data between modern (Mathieson et al., 2023) and ancient (Mathieson et al., 2015) Europeans shows that at the FADS1/2 locus (biosynthesis of ω-3 and ω-6 lipids), directional selection has continued for several thousand years. A yet unknown role of the melanocortin 1 receptor (MC1R) gene in reproductive biology has been discovered. Its effect on the number of offspring was stronger in women. Although variants of this gene determine ~73 % of the heritability of the red hair color, phenotypically the red color in the study population was not associated with NEB. After exclusion of red-haired women from the analysis, observed MC1R effect on the number of offspring in the British Biobank persisted. No relation was detected between effects of specific SNVs on hair color and on NEB. Intron insertion/deletion polymorphism of the CADM2 gene manifested the strongest association with childlessness. For this gene, which codes for a cell adhesion molecule and is expressed in the brain, a strong balancing selection signal and an association with risky behavior have been shown (Boutwell et al., 2017). A female-specific association with childlessness was found for transcription regulator gene PPP3R1 (Mathieson et al., 2023). The results obtained in that paper were validated for 35 identified loci in a sample of 34,367 FinnGen women (Mathieson et al., 2023). None of the signals identified in that study showed significant genome-wide associations with educational attainment, church attendance, or indices of social deprivation. In an assessment of potential functional significance of the found variants, it was noted that most of the found signals of modern directional selection are related to the hypothalamic–pituitary–gonadal axis, which regulates fertility and reproductive aging.

In several articles, authors separately studied the relation between educational attainment and the NEB. In a sample of 129,808 Icelanders born between 1910 and 1990, the POLYEDU polygenic index of educational attainment showed a negative correlation with the NEB, with gradual diminution in several observed generations (Kong et al., 2017). In particular, an increase in the frequency of the rs62056842 variant in an intron of the MAPT gene, which is expressed in the nervous system and is associated with reduced educational attainment, was detected. When children born to mothers aged 21 or younger (18 % of all children in the sample) and children born to men aged 22 or younger (13 %) were excluded from the analysis, the correlations disappeared (Kong et al., 2017).

Another study points to a borderline inverse correlation between the NEB and the polygenic educational attainment score among men, with the number of children regressing positively in the interaction of income and the PolyEduc polygenic score, thus indicating a positive correlation between income and the number of offspring in men with genetic predisposition to higher education (Fieder, Huber, 2022). In an analysis of the association of the polygenic score of fertility with 33 polygenic traits in two generations of Europeans from the British Biobank (348,595 people of European descent, taking into account the number of siblings and the number of offspring), it was shown that polygenic scores that predict higher income and education attainment are correlated with reduced fertility, whereas polygenic risk scores for ADHD, depressive disorder, and coronary heart disease as well as a higher BMI and extraversion predict more offspring (Hugh- Jones, Abdellaoui, 2022). That paper indicates that educational attainment and the risk of ADHD and a depressive disorder are selected among young mothers (age at first birth before 22), but the natural selection was reversed among older mothers. On the other hand, several anthropometric polygenic scores are selected only among older ones. By contrast, the largest analysis in terms of sample size (Mathieson et al., 2023) did not reveal selection against educational attainment.

In modern society, out of 15 categories of genes that determine phenotypic traits, those responsible for metabolism, nutritional habits, psychiatric indicators, dermatological signs (in men), social cognition, and reproduction correlate with the NEB (Song et al., 2021).

A positive correlation between polygenic risk scores for ADHD and fertility was also demonstrated in a comparison of a sample of 20,183 people with ADHD and a control sample of 35,191 residents of Europe and the United States (Demontis et al., 2019).

A limitation of genome-wide association studies is that they detect only a genetic locus, not a specific gene or polymorphic site associated with a given trait. Just as in a bioinformatic analysis, a high proportion of the associations falls into intergenic regions and cannot always be interpreted. A lot of controversy is caused by the stratification of a population sample; this approach can significantly affect the results (Sohail et al., 2019; Mills, Mathieson, 2022). Researchers emphasize the nonrandom compilation of cohorts as a source of bias because the very consent to participate in the study correlates, for example, with educational attainment not only in the frequently used American commercial sample 23andMe but also in the British Biobank (Mills, Mathieson, 2022; Schork et al., 2022). It is reported that a meta-analysis of small populations of Europeans from small geographic areas can give incorrect results due to the different proportions of ancestral populations of pastoralists, hunter-gatherers, and farmers who participated in the formation of the modern population of Europe in different regions; on the other hand, when examining large populations settled in different climatic regions, it is necessary to take into account the influence of climatic factors on the phenotype. In the UK, geographic clustering of genetic variants was found that affects complex traits, including alleles associated with educational attainment, thereby proving the influence of demographic factors on the correlation between genes and the environment (Abdellaoui et al., 2019). The vast majority of genome-wide association studies have been performed on populations of European origin, and therefore the findings cannot be automatically extrapolated to humanity as a whole; in addition, the number of studies with sufficient statistical power is small.

## Analysis of frequencies of genetic variants in close generations

The direction of selection can be assessed by comparison of genotypes among people born in the same population during periods adjacent to significant natural, socioeconomic, or political events in the region of their birth. The difference in allele frequencies in this case may reflect increased perinatal, prenatal, and infant mortality or a difference in the number of children among reproductive-age people carrying different genotypes during the period of the events under study.

Similar work was done on data from the British Biobank (Wu et al., 2022). A genome-wide association study of infant mortality rates by place and year of birth was performed. Cohorts born between 1936 and 1970 in England and Wales experienced a decline of infant mortality with spikes during World War II. Several statistically significant loci were found, including missense variant rs1446585 of the R3HDM1 gene near the LCT gene and missense variant rs5743618 of the TLR1 gene as well as rs2852853 in an intron of 7-dehydrocholesterol reductase gene DHCR7 (vitamin D metabolism), rs9944197 in an intron of the gene of ribosomal protein EFL1, and intergenic rs10521293. Those authors were especially interested in the LCT and TLR1/6/10 loci, which had previously shown natural selection among Europeans (Mathieson et al., 2015). The frequency of these alleles did not differ by year of birth in regions with low infant mortality but differed in this manner in regions with high infant mortality. The biggest difference was noted in 1942 (a year after the maximum of the German bombing), but the density of the bombings by region did not match the level of infant mortality; accordingly, those authors attribute the observed effect to harsh living conditions and food shortages.

A work comparing genotypes among three groups of adolescents who born before, during, and after the socioeconomic crisis of the 1990s in Russia was previously published by us (Mikhailova et al., 2022). We analyzed frequencies of common genetic variants previously found to be associated with stress resistance and stress-induced disorders in populations of Novosibirsk city schoolchildren aged 14–17 years. A statistically significant increase in frequencies of stress-protective variant rs4680 G of the COMT gene and long (7R+8R) tandem repeats in exon 3 of the DRD4 gene was found in the “stress” group. Both genes belong to the dopaminergic regulatory system. We hypothesized that under the conditions of prolonged social stress, carriers of certain genotypes have more offspring due to better adaptation to the conditions of socioeconomic deprivation because social stress affects fertility.

A limitation of such research is that alterations of allele frequencies occur within some short periods and may not affect the genetic pool of the population, especially owing to a drop of the birth rate in populations during of major destructive events. Furthermore, it is difficult to take into consideration all the factors that can potentially alter allele frequencies.

## Conclusion

Despite the limitations of each approach and the lack of information about directional selection in African and Asian populations, several dozen genetic variants and a number of polygenic traits have been found that have undergone natural selection during human evolution. Genetic loci and phenotypic traits have been identified whose direction of natural selection has not changed from the Neolithic to our time, although the intensity of the selection has varied (LCT and FAD1/2). For some genetic variants, the direction of adaptation changed, probably as a result of an encounter with pathogens (e. g., rs34536443 of the TYK2 gene). In modern populations, there has been a reversal of directional selection relative to previous evolution for polygenic scores of height in women and for the BMI in men. A considerable number of genetic variants that are reported to be associated with inflammatory diseases (including Crohn’s disease and atopic dermatitis) have been positively selected in the past, but it is not clear whether these variants are now under selection pressure or the observed genotype ratio is already a consequence of balancing selection due to antagonistic pleiotropy. Conflicting data have been obtained about selection of relatively recent complex polygenic traits: income and educational attainment. It has been shown that the targets of selection – to a greater extent than in previous centuries – are genes responsible for social adaptation and behavioral phenotypes. For instance, the positive association of the ADHD polygenic risk score with fertility as documented by several researchers is indicative of selection for this phenotype in modern populations.

## Conflict of interest

The authors declare no conflict of interest.

## References

Abdellaoui A., Hugh-Jones D., Yengo L., Kemper K.E., Nivard M.G.,
Veul L., Holtz Y., Zietsch B.P., Frayling T.M., Wray N.R., Yang J.,
Verweij K.J.H., Visscher P.M. Genetic correlates of social stratification
in Great Britain. Nat. Hum. Behav. 2019;3(12):1332-1342. DOI
10.1038/s41562-019-0757-5.

Abondio P., Cilli E., Luiselli D. Inferring signatures of positive selection
in whole-genome sequencing data: an overview of haplotype-based
methods. Genes. 2022;13(5):926. DOI 10.3390/genes13050926

Arkhangelskiy V.N., Shulgin S.G., Zinkina Yu.V. Reproductive behavior
of Russian women as depending on their level of education.
Vestnik Rossiyskogo Universiteta Druzhby Narodov. Seriya: Sotsiologiya
= RUDN Journal of Sociology. 2020;20(3):546-559. DOI
10.22363/2313-2272-2020-20-3-546-559. (in Russian)

Arkhangelskiy V.N., Rostovskaya T.K., Vasilieva E.N. Influence of the
standard of living on the reproductive behavior of Russians: gender
aspect. Zhenshchina v Rossiyskom Obshchestve = Woman in Russian
Society. 2021;Spec.iss.:3-24. DOI 10.21064/WinRS.2021.0.1.
(in Russian)

Arner A.M., Grogan K.E., Grabowski M., Reyes-Centeno H., Perry
G.H. Patterns of recent natural selection on genetic loci associated
with sexually differentiated human body size and shape phenotypes.
PLoS Genet. 2021;17(6):e1009562. DOI 10.1371/journal.
pgen.1009562.

Balbo N., Billari F.C., Mills M. Fertility in advanced societies: a review
of research: La fécondité dans les sociétés avancées: un examen des
recherches. Eur. J. Popul. 2013;29(1):1-38. DOI 10.1007/s10680-
012-9277-y.

Barban N., Jansen R., de Vlaming R., Vaez A., Mandemakers J.J.,
Tropf F.C., Shen X., Wilson J.F., Chasman D.I., Nolte I.M., …
Lee J.J., Benjamin D.J., Cesarini D., Koellinger P.D., den Hoed M.,
Snieder H., Mills M.C. Genome-wide analysis identifies 12 loci influencing
human reproductive behavior. Nat. Genet. 2016;48(12):
1462-1472. DOI 10.1038/ng.3698.

Beauchamp J.P. Genetic evidence for natural selection in humans in
the contemporary United States. Proc. Natl. Acad. Sci. USA. 2016;
113(28):7774-7779. DOI 10.1073/pnas.1600398113.

Boutwell B., Hinds D.; 23andMe Research Team; Tielbeek J., Ong K.K.,
Day F.R., Perry J.R.B. Replication and characterization of CADM2 and MSRA genes on human behavior. Heliyon. 2017;3(7):e00349.
DOI 10.1016/j.heliyon.2017.e00349

Byars S.G., Ewbank D., Govindaraju D.R., Stearns S.C. Colloquium
papers: natural selection in a contemporary human population. Proc.
Natl. Acad. Sci. USA. 2010;107(Suppl.1):1787-1792. DOI 10.1073/
pnas.0906199106.

Charpin D., Gouitaa M. Why is the prevalence of allergic diseases increasing?
A critical assessment of some classical risk factors. Mediators
Inflamm. 2001;10(6):292-294. DOI 10.1080/096293501527
00920.

Cohen A., Dehejia R., Romanov D. Financial incentives and fertility.
Rev. Econ. Stat. 2013;95(1):1-20. DOI 10.1162/REST_a_00342.

Demontis D., Walters R.K., Martin J., Mattheisen M., Als T.D., Agerbo
E., Baldursson G., Belliveau R., Bybjerg-Grauholm J., Bækvad-
Hansen M., … Werge T., Mors O., Mortensen P.B., Daly M.J., Farao-ne
S.V., Børglum A.D., Neale B.M. Discovery of the first genomewide
significant risk loci for attention deficit/hyperactivity disorder.
Nat. Genet. 2019;51(1):63-75. DOI 10.1038/s41588-018-0269-7.

Fieder M., Huber S. Contemporary selection pressures in modern societies?
Which factors best explain variance in human reproduction
and mating? Evol. Hum. Behav. 2022;43(1):16-25. DOI 10.1016/
j.evolhumbehav.2021.08.001.

Field Y., Boyle E.A., Telis N., Gao Z., Gaulton K.J., Golan D., Yengo
L., Rocheleau G., Froguel P., McCarthy M.I., Pritchard J.K. Detection
of human adaptation during the past 2000 years. Science.
2016;354(6313):760-764. DOI 10.1126/science.aag0776.

Grossman S.R., Andersen K.G., Shlyakhter I., Tabrizi S., Winnicki S.,
Yen A., Park D.J., Griesemer D., Karlsson E.K., Wong S.H., Cabili
M., Adegbola R.A., Bamezai R.N., Hill A.V., Vannberg F.O.,
Rinn J.L.; 1000 Genomes Project; Lander E.S., Schaffner S.F., Sabeti
P.C. Identifying recent adaptations in large-scale genomic data.
Cell. 2013;152(4):703-713. DOI 10.1016/j.cell.2013.01.035.

Hugh-Jones D., Abdellaoui A. Human capital mediates natural selection
in contemporary humans. Behav. Genet. 2022;52(4-5):205-234.
DOI 10.1007/s10519-022-10107-w.

Kerner G., Laval G., Patin E., Boisson-Dupuis S., Abel L., Casanova
J.L., Quintana-Murci L. Human ancient DNA analyses reveal the
high burden of tuberculosis in Europeans over the last 2,000 years.
Am. J. Hum. Genet. 2021;108(3):517-524. DOI 10.1016/j.ajhg.
2021.02.009.

Klunk J., Vilgalys T.P., Demeure C.E., Cheng X., Shiratori M.,
Madej J., Beau R., Elli D., Patino M.I., Redfern R., DeWitte S.N.,
Gamble J.A., Boldsen J.L., Carmichael A., Varlik N., Eaton K.,
Grenier J.C., Golding G.B., Devault A., Rouillard J.M., Yotova V.,
Sindeaux R., Ye C.J., Bikaran M., Dumaine A., Brinkworth J.F.,
Missiakas D., Rouleau G.A., Steinrücken M., Pizarro-Cerdá J.,
Poinar H.N., Barreiro L.B. Evolution of immune genes is associated
with the Black Death. Nature. 2022;611(7935):312-319. DOI
10.1038/s41586-022-05349-x.

Kong A., Frigge M.L., Thorleifsson G., Stefansson H., Young A.I.,
Zink F., Jonsdottir G.A., Okbay A., Sulem P., Masson G., Gudbjartsson
D.F., Helgason A., Bjornsdottir G., Thorsteinsdottir U., Stefansson
K. Selection against variants in the genome associated with
educational attainment. Proc. Natl. Acad. Sci. USA. 2017;114(5):
E727-E732. DOI 10.1073/pnas.1612113114

Kuijpers Y., Domínguez-Andrés J., Bakker O.B., Gupta M.K., Grasshoff
M., Xu C.J., Joosten L.A.B., Bertranpetit J., Netea M.G., Li Y.
Evolutionary trajectories of complex traits in European populations
of modern humans. Front. Genet. 2022;13:833190. DOI 10.3389/
fgene.2022.833190.

Lewandowska M., Jędrychowska-Dańska K., Płoszaj T., Witas P.,
Zamerska A., Mańkowska-Pliszka H., Witas H.W. Searching for
signals of recent natural selection in genes of the innate immune
response – ancient DNA study. Infect. Genet. Evol. 2018;63:62-72.
DOI 10.1016/j.meegid.2018.05.008

Marnetto D., Pankratov V., Mondal M., Montinaro F., Pärna K., Vallini
L., Molinaro L., Saag L., Loog L., Montagnese S., Costa R.;
Estonian Biobank Research Team; Metspalu M., Eriksson A., Pagani
L. Ancestral genomic contributions to complex traits in contemporary
Europeans. Curr. Biol. 2022;32(6):1412-1419.e3. DOI
10.1016/j.cub.2022.01.046.

Mathieson I., Terhorst J. Direct detection of natural selection in
Bronze Age Britain. Genome Res. 2022;32(11-12):2057-2067. DOI
10.1101/gr.276862.122.

Mathieson I., Lazaridis I., Rohland N., Mallick S., Patterson N.,
Roodenberg S.A., Harney E., Stewardson K., Fernandes D., Novak
M., Sirak K., Gamba C., Jones E.R., Llamas B., Dryomov S.,
Pickrell J., Arsuaga J.L., de Castro J.M., Carbonell E., Gerritsen F.,
Khokhlov A., Kuznetsov P., Lozano M., Meller H., Mochalov O.,
Moiseyev V., Guerra M.A., Roodenberg J., Vergès J.M., Krause J.,
Cooper A., Alt K.W., Brown D., Anthony D., Lalueza-Fox C.,
Haak W., Pinhasi R., Reich D. Genome-wide patterns of selection
in 230 ancient Eurasians. Nature. 2015;528(7583):499-503. DOI
10.1038/nature16152.

Mathieson I., Day F.R., Barban N., Tropf F.C., Brazel D.M.; eQTLGen
Consortium; BIOS Consortium; Vaez A., van Zuydam N., Bitarello
B.D., … Zhao W., Zhao Y., Snieder H., den Hoed M., Ong K.K.,
Mills M.C., Perry J.R.B. Genome-wide analysis identifies genetic
effects on reproductive success and ongoing natural selection at the
FADS locus. Nat. Hum. Behav. 2023;7(5):790-801. DOI 10.1038/
s41562-023-01528-6.

Mikhailova S.V., Ivanoshchuk D.E., Yushkevich E.A., Bairqdar A.,
Anisimenko M.S., Shcherbakova L.V., Denisova D.V., Orlov P.S.
Prevalence of common alleles of some stress resilience genes among
adolescents born in different periods relative to the socioeconomic
crisis of the 1990s in Russia. Curr. Issues Mol. Biol. 2022;45(1):51-
65. DOI 10.3390/cimb45010004.

Mills M.C., Mathieson I. The challenge of detecting recent natural
selection in human populations. Proc. Natl. Acad. Sci. USA. 2022;
119(15):e2203237119. DOI 10.1073/pnas.2203237119.

Mitteroecker P., Huttegger S.M., Fischer B., Pavlicev M. Cliff-edge
model of obstetric selection in humans. Proc. Natl. Acad. Sci. USA.
2016;113(51):14680-14685. DOI 10.1073/pnas.1612410113.

Palamara P.F., Terhorst J., Song Y.S., Price A.L. High-throughput inference
of pairwise coalescence times identifies signals of selection
and enriched disease heritability. Nat. Genet. 2018;50(9):1311-1317.
DOI 10.1038/s41588-018-0177-x.

Pankratov V., Yunusbaeva M., Ryakhovsky S., Zarodniuk M.; Estonian
Biobank Research Team; Yunusbayev B. Prioritizing autoimmunity
risk variants for functional analyses by fine-mapping mutations
under natural selection. Nat. Commun. 2022;13(1):7069. DOI
10.1038/s41467-022-34461-9.

Raj T., Kuchroo M., Replogle J.M., Raychaudhuri S., Stranger B.E.,
De Jager P.L. Common risk alleles for inflammatory diseases are
targets of recent positive selection. Am. J. Hum. Genet. 2013;92(4):
517-529. DOI 10.1016/j.ajhg.2013.03.001.

Reeve C.L., Heeney M.D., Woodley of Menie M.A. A systematic review
of the state of literature relating parental general cognitive ability
and number of offspring. Pers. Individ. Differ. 2018;134:107-
118. DOI 10.1016/j.paid.2018.05.036.

Saklayen M.G. The global epidemic of the metabolic syndrome.
Curr. Hypertens. Rep. 2018;20(2):12. DOI 10.1007/s11906-018-
0812-z.

Sanjak J.S., Sidorenko J., Robinson M.R., Thornton K.R., Visscher P.M.
Evidence of directional and stabilizing selection in contemporary
humans. Proc. Natl. Acad. Sci. USA. 2018;115(1):151-156. DOI
10.1073/pnas.1707227114.

Schork A.J., Peterson R.E., Dahl A., Cai N., Kendler K.S. Indirect paths
from genetics to education. Nat. Genet. 2022;54(4):372-373. DOI
10.1038/s41588-021-00999-5.

Sohail M., Maier R.M., Ganna A., Bloemendal A., Martin A.R.,
Turchin M.C., Chiang C.W., Hirschhorn J., Daly M.J., Patterson N.,
Neale B., Mathieson I., Reich D., Sunyaev S.R. Polygenic adaptation
on height is overestimated due to uncorrected stratification in genome-wide association studies. eLife. 2019;8:e39702. DOI
10.7554/eLife.39702.

Song W., Shi Y., Wang W., Pan W., Qian W., Yu S., Zhao M., Lin G.N.
A selection pressure landscape for 870 human polygenic traits.
Nat. Hum. Behav. 2021;5(12):1731-1743. DOI 10.1038/s41562-
021-01231-4.

Speidel L., Forest M., Shi S., Myers S.R. A method for genome-wide
genealogy estimation for thousands of samples. Nat. Genet. 2019;
51(9):1321-1329. DOI 10.1038/s41588-019-0484-x.

Suvorov A. Population numbers and reproductive health. Endocrinology.
2021;162(11):bqab154. DOI 10.1210/endocr/bqab154.

Testa M.R. On the positive correlation between education and fertility
intentions in Europe: individual- and country-level evidence.
Adv. Life Course Res. 2014;21:28-42. DOI 10.1016/j.alcr.2014.01.
005.

Tropf F.C., Stulp G., Barban N., Visscher P.M., Yang J., Snieder H.,
Mills M.C. Human fertility, molecular genetics, and natural selection
in modern societies. PLoS One. 2015;10(6):e0126821. DOI
10.1371/journal.pone.0126821.

Turner N., Robbins K. Association between county-level natality and
income in the US, 2000–2020. JAMA Pediatr. 2023;177(2):198-202.
DOI 10.1001/jamapediatrics.2022.4814.

Wolf E., Sonenklar N., Schefft M., Haskell H., James J. Is there evidence
of ADHD overdiagnosis in children? Am. Fam. Physician.
2023;107(3):292-296.

Wu Y., Furuya S., Wang Z., Nobles J.E., Fletcher J.M., Lu Q. GWAS on
birth year infant mortality rates provides evidence of recent natural
selection. Proc. Natl. Acad. Sci. USA. 2022;119(12):e2117312119.
DOI 10.1073/pnas.2117312119.

Yasumizu Y., Sakaue S., Konuma T., Suzuki K., Matsuda K., Murakami
Y., Kubo M., Palamara P.F., Kamatani Y., Okada Y. Genomewide
natural selection signatures are linked to genetic risk of modern
phenotypes in the Japanese population. Mol. Biol. Evol. 2020;
37(5):1306-1316. DOI 10.1093/molbev/msaa005.

Zeidan J., Fombonne E., Scorah J., Ibrahim A., Durkin M.S., Saxena
S., Yusuf A., Shih A., Elsabbagh M. Global prevalence of autism:
a systematic review update. Autism Res. 2022;15(5):778-790. DOI
10.1002/aur.2696.

